# The Novelty of Icosapent Ethyl in the Management of Hypertriglyceridemia and Alleviating Cardiovascular Risk

**DOI:** 10.1155/2021/6696915

**Published:** 2021-01-12

**Authors:** Muhammad Shoaib Khan, Muhammad Ishaq, Muhammad Talha Ayub, Ateeq U. Rehman, John J. Hayes, Mohammad Mortada, Robert W. W. Biederman

**Affiliations:** ^1^Department of Internal Medicine, Marshfield Clinic Health System, Marshfield, Wisconsin, USA; ^2^Department of Cardiovascular Diseases, Rush University, Chicago, Illinois, USA; ^3^Department of Cardiovascular Diseases, Marshfield Clinic Health System, Marshfield, Wisconsin, USA; ^4^Department of Clinical Cardiac Electrophysiology, Aurora Health Care, Milwaukee, Wisconsin, USA; ^5^Division of Cardiac MRI, Allegheny General Hospital, Pittsburgh, Pennsylvania, USA

## Abstract

Hypertriglyceridemia is believed to be independently associated with an elevated risk of cardiovascular disease (CVD) events. Lifestyle changes and dietary modifications are recommended for individuals with high serum triglyceride (TG) levels (150-499 mg/dl), and pharmacological therapy in addition to lifestyle modification is recommended when serum TG levels ≥ 500 mg/dl. A residual cardiovascular risk remains even in statin appropriate treated patients with CVD risk factors, and in this patient population, hypertriglyceridemia poses an independent and increased risk of ischemic events. In December 2019, the US FDA approved icosapent ethyl (IPE) as an adjunct to a maximally tolerated statin to reduce the risk of CVD events in adults with serum triglycerides > 150 mg/dl and have either established cardiovascular disease or diabetes and two or more additional CVD risk factors. Since IPE significantly decreases total ischemic events in the aforementioned patient population, it would be intriguing to know whether IPE alone added an advantage to lifestyle modification in the low-risk population, who has serum triglyceride between 150 mg/dl and 499 mg/dl.

## 1. Introduction

Cardiovascular disease (CVD) is believed to be the major cause of preventable death in the US [1]. Hypertriglyceridemia promotes the formation of atherogenic LDL-C and decreases cholesterol clearance from circulation by reducing HDL-C mediated transport to the liver [2]. Hypertriglyceridemia is a risk factor for CVD, and several studies and analysis in the past hinted at increased risk of CVD events with a higher blood level of triglycerides [3, 4]. Regardless of blood levels of other lipid components, elevated serum triglyceride (TG) levels are believed to be independently associated with an elevated risk of CVD events [5, 6]. It has been observed in prior studies that mutations in the lipoprotein lipase (LPL) gene predispose to hypertriglyceridemia, conferring increased risk of CVD [7–11]. Moreover, it has also been observed that mutations in genes (such as APOC3, ANGPTL3, and ANGPTL4) acting as negative regulators of LPL decrease serum triglyceride levels and, therefore, alleviate CVD risk [7–11]. However, a recent study has suggested that the decrease in triglyceride levels in Mendelian randomization studies may not be the key variable but rather a marker [12]. In that study, the absolute reduction in ApoB was thought to be more predictive of reduced CVD risk comparing to reduction in serum TG level and/or LDL-C. The changes in serum TG levels are associated with changes in ApoB levels, an indicator of the number of atherogenic particles (chylomicrons, VLDL, remnants, IDL, LDL, and Lp (a)). Thus, it may not be the triglycerides by itself but rather that increased triglyceride levels leads to an increase in atherogenic particles and therefore enhanced CVD risk.

Fasting serum TG level of less than 150 milligrams per deciliter (mg/dl) is regarded as normal [13]. Traditionally, first-line therapy for individuals with high serum TG levels (150-499 mg/dl) involves lifestyle changes such as dietary modification, weight loss, minimizing the use of refined sugars, refined grains, white bread, bagels, fried food, decreased alcohol consumption, and increased physical activity [14–17]. When the serum TG levels are ≥500 mg/dl, pharmacological therapy (with fibrates, niacin, omega-3 fatty acid, and statins) is recommended in addition to lifestyle intervention [13, 14].

### 1.1. New Era of Hypertriglyceridemia Treatment While Being on Statin Therapy

Studies have shown that even in statin appropriate treated patients with CVD risk factors (such as smoking, lack of exercise, obesity, hypertension, dyslipidemia, family history of heart disease, and aging), a sizeable amount of cardiovascular risk persists [18]. It is of no surprise that several studies have shown that in such an aforementioned patient population hypertriglyceridemia poses an independent and increased risk of ischemic events [19–22]. Commonly used TG-lowering medications such as fibrates and niacin have not been proven to show a distinct reduction in CVD events in statin-treated patients [23]. This has posed a significant challenge to the physicians in the past to address the residual cardiovascular risk despite being on a maximally tolerated statin. Not until recently, we found a solution to this problem. In December 2019, the US FDA approved icosapent ethyl (IPE) as an adjunct to a maximally tolerated statin to reduce the risk of CVD events in adults with serum triglycerides > 150 mg/dl and have either established cardiovascular disease or diabetes and two or more additional CVD risk factors [24].

### 1.2. Icosapent Ethyl and REDUCE-IT Trial

**Figure 1:**
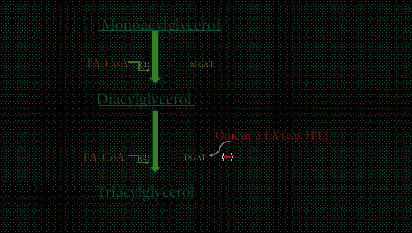
Triglyceride synthesis pathway and mechanism of action of omega-3 FA (fatty acid): In step (1), monoacylglycerol and FA-CoA combine to form diacylglycerol. This step is mediated by the MGAT enzyme. In step (2), diacylglycerol combine with another FA-CoA to form triacylglycerol, final step in triglyceride synthesis pathway. This last step is mediated by DGAT enzyme. This enzyme is inhibited by omega-3 FA, including IPE. Abbreviations: FA-CoA: fatty acyl CoA; MGAT: monoacylglycerol acyltransferase; DGAT: diacylglycerol acyltransferase enzyme; IPE: icosapent ethyl.

IPE is an ultrapure omega-3 fatty acid product and is a prescription form of ethyl eicosapentaenoic acid (EPA). Possible mechanisms by which IPE (and other omega-3 fatty acids) decreases serum TG may involve reducing substrate (fatty acid) availability and decreasing the activity of diacylglycerol acyltransferase [25], thus, decreasing TG synthesis (Figure 1). For years, it has been approved by the US Food as an adjunct to diet to reduce TG levels in adult patients with serum TG level ≥ 500 mg/dl [26]. However, its beneficial effects in reducing CVD risk for patients already on maximally tolerated statin therapy was recently established.

One of the prior studies (JELIS trial) compared a combination of statin therapy and pure eicosapentaenoic acid (EPA) with statin therapy alone. The results were remarkable and in comparison to low-intensity statin therapy alone, a 19% relative risk reduction in cardiovascular events with combination therapy was observed [27]. However, the results of this trial had certain limitations such as the trial being an open-label design, study population involving mainly people of Japanese ethnicity, and lack of placebo control.

Another large-scale, global, randomized, double-blind, placebo-controlled trial (STRENGTH trial) evaluated whether Epanova (a modified formulation of omega-3 fatty acid) 4 g daily reduced the rate of cardiovascular events in statin-treated patients with hypertriglyceridemia and low levels of HDL-C comparing to placebo (corn oil) [28, 29]. The study population had serum TG ≥ 180 to <500 mg/dl and HDL‐C < 42 mg/dl (men) or <47 mg/dl (women). Further, they had high-risk factors for cardiovascular events. These included established atherosclerotic cardiovascular disease, diabetes with one additional risk factor (such as cigarette smoking, hypertension, high CRP, or microalbuminuria) and had high-risk primary prevention (age > 50 years for men, >60 years for women, family history of premature CAD, cigarette smoking, CRP > 2.0 mg/l, deranged renal function, or coronary calcium score > 300 Agatston units. The primary efficacy outcome was time to the first event of myocardial infarction, cardiovascular death, stroke, coronary revascularization, or hospitalization for unstable angina. Among statin-treated patients at high cardiovascular risk, the addition of carboxylic acid formulation of omega-3 fatty acids (eicosapentaenoic acid and docosahexaenoic acid), compared with corn oil, resulted in no significant difference in a composite outcome of major adverse cardiovascular events. Further, the study results did not seem to favor the use of omega-3 fatty acid formulation to reduce major adverse cardiovascular events in high-risk patients. However, in this study, all patients had high risk of future cardiovascular events. It did not reveal whether there are potential benefits in a lower-risk population.

In contrast, REDUCE-IT was a multicenter, randomized, double-blind, placebo-controlled trial, which evaluated the effect of IPE on CVD outcomes in patients with hypertriglyceridemia (fasting serum TG levels of 135 to 499 mg/dl), already on a statin, and with either established CVD or diabetes with other cardiovascular risk factors [30]. Icosapent ethyl reduced the risk of the primary composite CVD endpoint of cardiovascular death, nonfatal myocardial infarction, nonfatal stroke, coronary revascularization, or unstable angina (17.2 versus 22.0 percent, hazard ratio 0.75, 95% CI 0.68-0.83) after a median follow-up of 4.9 years [30]. From baseline to one-year follow-up, the median triglyceride level decreased 18 percent in the intervention group and increased 2.2 percent in the control group [31]. Even though LDL-C levels increased in both groups (treatment group 3.1 percent, control group 10.2 percent) but the increase in the control group was more than 3 times the increase in the treatment group. This perhaps indicates that IPE may have a role in slowing down the rate of LDL-C increase with aging. As per one of the American Heart Association reports, the majority of CVD events and deaths occur in the elderly patient population [32]. LDL blood cholesterol levels increase with age [32, 33]. The mechanisms behind this age-related increase in plasma cholesterol are not well understood; however, one plausible explanation can be a gradual reduction in the fractional clearance of LDL from the circulation over time [34, 35].

The rates of new-onset atrial fibrillation were significantly higher in the treatment group comparing to the placebo group (5.3 versus 3.9 percent) and a relatively larger percentage of patients in the treatment group were hospitalized for atrial fibrillation or flutter (3.1% vs. 2.1%, *P* = 0.004) [31]. Serious bleeding events were reported in 2.7% of the patients in the intervention group comparing to 2.1% in the placebo group (*P* = 0.06); however, none of the groups had fatal major bleeding [31]. Nevertheless, the frequency of grave adverse events leading to the cessation of the trial drug was comparable in both the groups [31].

At two-year follow-up, C-reactive protein levels decreased by 13.9 percent in the treatment group and increased by 32.2 percent in the control group. A subsequent analysis REDUCE-IT results by multiple statistical models revealed that IPE significantly reduced the happening of first, subsequent, and total ischemic events [36]. In comparison, fibrates and niacin reduce serum TG; however, they have not shown a clear benefit in terms of reducing CVD events in patients receiving statin therapy [37–39].

### 1.3. Future of IPE

From REDUCE-IT trial results, one would think of IPE as a blessing drug as (when used in addition to statins) it can reduce the risk of the major CVD events and decrease serum C-reactive protein in addition to lowering serum TG levels. This benefit was observed in patient population, who had either established CVD or diabetes with 2 CVD risk factors. Traditionally, drug therapy as an adjunct to diet for predominant hypertriglyceridemia (regardless of CVD risk factors) treatment is considered when serum TG level ≥ 500 mg/dl [13–15]. The fact that regardless of blood levels of other lipid components, elevated serum triglyceride (TG) levels are believed to be independently associated with an elevated risk of CVD events [5, 6]. IPE may have a beneficial role even in patients with serum TG level between 150 and 499 mg/dl without established CVD or without DM with two additional CVD risk factors. This can be especially plausible when someone has hypertriglyceridemia but the other biomarkers and ASCVD risk predictors are not significant enough to qualify a patient for statins. The ASCVD risk calculator does not take into account serum triglyceride level as a distinct marker of CVD risk [40], and as a result, one may have higher serum TG and yet total cholesterol (and HDL) be not significantly abnormal. Similarly, it can be a plausible drug of choice for those patients who have hypertriglyceridemia but are intolerant to statins and can be preferred over niacin and fibrates.

However, as the incidence of hospitalization from new-onset atrial fibrillation/flutter was higher in the treatment group comparing to the placebo group, further studies involving a greater population and longer duration of follow-up are warranted to further clarify the cardioprotective mechanisms of IPE, as atrial fibrillation/flutter can pose an increased risk of future stroke events, especially if the AF episode lasts greater than 24 hours. Prior studies have suggested that even with (device detected) asymptomatic AF episodes lasting longer than 24 hours, the risk for stroke and systemic thromboembolism approaches that of clinically diagnosed AF [41, 42].

Moreover, it should be noted that in the STRENGTH trial the reduction in serum TG levels was similar to the reduction in TG levels seen in the REDUCE-IT trial. This suggests that the potential beneficial effects of reducing serum TG levels may not entirely be explained from lowering TG levels only. However, in REDUCE-IT trial, higher plasma and red blood cell levels of EPA were achieved compared with those in STRENGTH trial. It may be premature to state that this difference would completely account to different results observed in these aforementioned trials.

## 2. Conclusion

With an established benefit of IPE in reducing CVD events in patients with established CVD and/or DM with 2 additional CVD risk factors, who continue to have serum TG level greater than 150 mg/dl despite maximally tolerated statin, it may be deemed plausible to extend its use in a patient population who have predominant hypertriglyceridemia and do not have established CVD and/or DM with 2 additional CVD risk factors.

However, in this aforementioned patient population, further studies comparing the risks and benefits of lifestyle modification only as against IPE use only, or lifestyle modification and IPE use as against IPE use only to establish the upper hand of one intervention over the other in treating dyslipidemia with predominant hypertriglyceridemia in the range of 150 to 499 mg/dl need to be performed.

## Data Availability

The data used in this article are available from the corresponding author upon request.

## Abbreviations

AF: Atrial fibrillation
ApoB: Apolipoprotein B
CVD: Cardiovascular disease
DM: Diabetes mellitus
EPA: Eicosapentaenoic acid
FDA: Food and Drug Administration
IPE: Icosapent ethyl
LDL: Low-density lipoprotein
LPL: Lipoprotein lipase
mg/dl: Milligrams per deciliter
TG: Triglyceride.
